# Long non-coding RNAs modulate tumor microenvironment to promote metastasis: novel avenue for therapeutic intervention

**DOI:** 10.3389/fcell.2023.1164301

**Published:** 2023-06-13

**Authors:** Sana Khurshid Baba, Sadaf Khursheed Baba, Rashid Mir, Imadeldin Elfaki, Naseh Algehainy, Mohammad Fahad Ullah, Jameel Barnawi, Faisal H. Altemani, Mohammad Alanazi, Syed Khalid Mustafa, Tariq Masoodi, Ammira S. Alshabeeb Akil, Ajaz A. Bhat, Muzafar A. Macha

**Affiliations:** ^1^ Watson-Crick Centre for Molecular Medicine, Islamic University of Science and Technology, Awantipora, Kashmir, India; ^2^ Department of Microbiology, Sher-I-Kashmir Institute of Medical Science (SKIMS), Soura, Kashmir, India; ^3^ Department of Medical Lab Technology, Prince Fahd Bin Sultan Research Chair Faculty of Applied Medical Sciences, University of Tabuk, Tabuk, Saudi Arabia; ^4^ Department of Biochemistry, Faculty of Science, University of Tabuk, Tabuk, Saudi Arabia; ^5^ Department of Chemistry, Faculty of Science, University of Tabuk, Tabuk, Saudi Arabia; ^6^ Human Immunology Department, Research Branch, Sidra Medicine, Doha, Qatar; ^7^ Department of Human Genetics-Precision Medicine in Diabetes, Obesity, and Cancer Program, Sidra Medicine, Doha, Qatar

**Keywords:** cancer, metastasis, long non-coding RNAs, tumor microenvironment, anoikis resistance, metabolic reprogramming, immune modulation

## Abstract

Cancer is a devastating disease and the primary cause of morbidity and mortality worldwide, with cancer metastasis responsible for 90% of cancer-related deaths. Cancer metastasis is a multistep process characterized by spreading of cancer cells from the primary tumor and acquiring molecular and phenotypic changes that enable them to expand and colonize in distant organs. Despite recent advancements, the underlying molecular mechanism(s) of cancer metastasis is limited and requires further exploration. In addition to genetic alterations, epigenetic changes have been demonstrated to play an important role in the development of cancer metastasis. Long non-coding RNAs (lncRNAs) are considered one of the most critical epigenetic regulators. By regulating signaling pathways and acting as decoys, guides, and scaffolds, they modulate key molecules in every step of cancer metastasis such as dissemination of carcinoma cells, intravascular transit, and metastatic colonization. Gaining a good knowledge of the detailed molecular basis underlying lncRNAs regulating cancer metastasis may provide previously unknown therapeutic and diagnostic lncRNAs for patients with metastatic disease. In this review, we concentrate on the molecular mechanisms underlying lncRNAs in the regulation of cancer metastasis, the cross-talk with metabolic reprogramming, modulating cancer cell anoikis resistance, influencing metastatic microenvironment, and the interaction with pre-metastatic niche formation. In addition, we also discuss the clinical utility and therapeutic potential of lncRNAs for cancer treatment. Finally, we also represent areas for future research in this rapidly developing field.

## Introduction

Cancer poses a significant threat to improving life expectancy as it remains one of the leading causes of global deaths (Bray et al., 2021). The preliminary data show that it is the leading cause of mortality in people before the age of 70 years in most countries worldwide. Since the last decade, cancer incidence and mortality rates have risen sharply around the globe with 19.3 million new cases and 10 million cancer deaths worldwide in 2020 (Sung et al., 2021). It is estimated that more than 1,670 people will die of cancer every day in the United States by 2023 (Siegel et al., 2023).

Metastasis, the spread of cancer cells from the primary site to distant organs, is a complex and multi-stage process that is the leading cause of cancer-related deaths. This process involves several key stages, which include local invasion, in which cancer cells invade surrounding tissues, and intravasation, in which cancer cells enter the bloodstream (Hanahan and Weinberg, 2011). The cancer cells then travel to distant sites, where they undergo extravasation into the surrounding tissues and form micro-metastatic colonies. Finally, these colonies can proliferate and become clinically identifiable macro-metastases, referred to as “cancer colonization” (Talmadge and Fidler, 2010; Lambert et al., 2017; Klein, 2020). The molecular mechanisms underlying metastasis are intricate and not yet fully understood, but research into this issue continues to evolve. This complexity highlights the need for new perspectives and approaches in the study of metastasis in order to better understand and ultimately combat this devastating aspect of cancer.

Ribonucleic acids (RNAs) play a critical role in the origin of life due to their unique properties as both catalysts and genetic materials (Higgs and Lehman, 2015). The “RNA world hypothesis” posits that early life on Earth was characterized by self-replicating RNA molecules before the evolution of proteins and DNA (Mercer et al., 2009). Despite the fact that only 2% of human RNA transcripts are translated into proteins, the remaining transcripts have been shown to play important regulatory functions through recent advancements in genomic technologies (Djebali et al., 2012). These transcripts, which do not code for proteins, are known as non-coding RNAs (ncRNAs) (Jarroux et al., 2017) and are divided into two main categories: regulatory and structural ncRNAs. Structural ncRNAs include ribosomal RNA (rRNA) and transfer RNA (tRNA), while regulatory ncRNAs are further divided into small non-coding RNAs (sncRNAs) and long non-coding RNAs (lncRNAs) based on their size (Esteller, 2011; Quinn and Chang, 2016). The most well-studied classes of sncRNAs are piwi-interacting RNAs (piRNAs), micro-RNAs (miRNAs), and endogenous small interfering RNAs (siRNAs) (Naqvi et al., 2009). These small non-coding RNAs play critical roles in the regulation of gene expression, which includes the repression of unwanted transcripts and the modulation of mRNA stability. Further research into the role of ncRNAs, particularly sncRNAs, in the regulation of gene expression holds the potential to reveal new insights into the biology of cells and pathogenesis of diseases.

Long non-coding RNAs (lncRNAs) are RNA transcripts longer than 200 nucleotides without the potential to code for proteins (Ming et al., 2021). These lncRNAs are usually located in the nucleus and are found in different nuclear compartments such as the chromatin and nucleoplasm. They play important roles in modulating nuclear organization and function (Yao et al., 2019). Some lncRNAs can be found in the cytoplasm (Wang and Chang, 2011; Chen, 2016). Based on their relative position to their proximate protein-coding genes (PCGs), lncRNAs are classified (Harrow et al., 2012; Hrdlickova et al., 2014) as (a) sense lncRNAs, which are within the PCG spanning multiple introns or exons; (b) antisense lncRNAs, which are transcribed from the opposite strand of a PCG; (c) intronic lncRNAs are found in the sense strand of an intron of a coding gene; (d) intergenic lncRNAs, which are transcripts located between two PCGs; and (**e**) bidirectional lncRNAs that are located on the opposite strand but within 1 kb of the promoter on the sense strand and are transcribed on the sense strand in the opposite direction to that of the promoter (Bär et al., 2016). The biological functions of lncRNAs can be classified into several categories, such as guides, scaffolds, decoys, and platforms (Wong et al., 2018). In addition, the expression of other genes is usually modulated by signals from lncRNAs (Grossi et al., 2020). As guides, lncRNAs can direct regulatory proteins such as transcription factors and epigenetic regulators to specific regions of the genome. As decoys, lncRNAs can modulate gene expression by binding with miRNAs, thereby blocking their ability to regulate target genes (Zhang et al., 2018). As scaffolds, lncRNAs can provide a platform for the assembly of protein complexes involved in various cellular processes (Zhang et al., 2019a). Finally, as platforms, lncRNAs can recruit various molecules through binding with different structural domains (Düzgün Ş et al., 2017). These various roles of lncRNAs highlight their importance in regulating gene expression and cellular processes, particularly in cancer.

For many years, researchers have suspected that lncRNAs could play a role in cancer but lacked concrete evidence to support this hypothesis (Rainier et al., 1993). However, with advancements in cancer transcriptome profiling and accumulating evidence that support lncRNA function, a number of differentially expressed lncRNAs have now been associated with cancer (Gibb et al., 2011). Importantly, several lncRNAs have been identified to be involved in different stages of the metastatic cascade, which includes invasion, intravasation, and extravasation. For example, the metastasis-associated lung adenocarcinoma transcript 1 (MALAT1) lncRNA was first identified as having a potential role in cancer during a comparative screening of non–small-cell lung cancer patients with and without metastatic tumors (Ji et al., 2003). This lncRNA is widely expressed in normal tissues in human (Ji et al., 2003; Hutchinson et al., 2007) and is found to be upregulated in a variety of cancers of the breast, prostate, colon, liver, and uterus in humans (Luo et al., 2006; Yamada et al., 2006; Lin et al., 2007; Guffanti et al., 2009). MALAT1 has been shown to interact with the protein SFPQ to promote the invasion and migration of colorectal cancer cells (Ji et al., 2014). One way in which lncRNAs contribute to metastasis is by regulating the expression of genes that are involved in metastatic processes. For example, the lncRNA HOX antisense intergenic RNA (HOTAIR), a 2.2-kb gene located in the mammalian HOXC locus on chromosome 12q13.13 (Rinn et al., 2007), has been shown to promote metastasis in breast cancer (BC) by repressing the expression of genes involved in cell adhesion and promoting the expression of genes involved in cell migration and invasion (Abba et al., 2021). This lncRNA was found to be highly upregulated in metastatic breast tumors, demonstrating up to 2000 times increased transcription over normal BC (Gupta et al., 2010). High levels of HOTAIR expression were found to be correlated with both metastasis and poor survival rate, linking this lncRNA with cancer invasiveness and patient prognosis (Gupta et al., 2010). lncRNAs also play an important role in regulating the epithelial–mesenchymal transition (EMT) process, which is crucial for the migration of cancer cells from the primary site to other parts of the body (Nieto et al., 2016; Brabletz et al., 2018; Nisar et al., 2021a). lncRNAs have been found to regulate important signaling pathways involved in EMT and are therefore considered key regulators of tumor metastasis (Guo et al., 2014; Jia et al., 2016; Heery et al., 2017). In particular, lncRNA H19, upregulated in a number of human cancers, such as in hepatocellular, bladder, and breast carcinomas (Berteaux et al., 2005; Barsyte-Lovejoy et al., 2006; Matouk et al., 2007), has been implicated in inducing EMT and cancer metastasis. This lncRNA has been shown to promote EMT in hepatocellular carcinoma by upregulating the expression of the transcription factor Snail (Wen et al., 2020; Yuan et al., 2021). In addition to these, multiple cancer–associated lncRNAs has been shown to regulate cancer invasion and metastasis (Raveh et al., 2015; Luo et al., 2018; Tang et al., 2018). Overall, many studies have suggested that lncRNAs are important regulators of cancer metastasis (Hunter et al., 2018; Slack and Chinnaiyan, 2019; Williams et al., 2019; Li et al., 2020a; Qin et al., 2020) and therefore serve as important therapeutic targets and disease biomarkers (Liu et al., 2021).

Metabolic reprogramming is a hallmark of cancer (Hanahan and Weinberg, 2011), which involves alterations in cellular metabolism to support the energy and biosynthetic demands of rapidly dividing cancer cells. Recent studies have shown that lncRNAs can modulate metabolic pathways and contribute to the metabolic reprogramming that occurs in cancer cells during metastasis. lncRNAs are involved in regulating the interplay between molecular signaling and metabolic reprogramming (Sellitto et al., 2021). This regulation is achieved through changes in cell metabolic processes during different stages of metastasis and by providing energy and essential metabolites for the continuous growth and proliferation of cancer cells (Phan et al., 2014). One example is the lncRNA MALAT1, which has been shown to promote metabolic reprogramming in BC cells by regulating the expression of genes involved in glycolysis and oxidative phosphorylation (Agostini et al., 2022). MALAT1 also enhances the Warburg effect by increasing lactate production and decreasing mitochondrial respiration, which in turn promotes the migration and invasion of multiple myeloma cells (Liu et al., 2020a). In addition, H19 has been shown to promote glycolysis in gastric cancer (GC) (Sun et al., 2021) and oral cancer (Yang et al., 2021) cells, thereby enhancing the Warburg effect and promoting the metastatic potential.

The tumor microenvironment (TME) regulates important tumor promotion and survival functions. Through a dynamic and multistep metastatic cascade, communication between the structural and cellular components of the TME permits cancer cells to disseminate from the primary site to distant areas and become invasive (Neophytou et al., 2021). On arrival at distant organ sites, metastatic cells form and interact with the TME, involving numerous processes like angiogenesis (De Palma et al., 2017), suppression and/or co-option of the innate and adaptive immune system (Kitamura et al., 2015; Karki and Kanneganti, 2019; Huntington et al., 2020), and the reprogramming of stromal populations to enable metastatic outgrowth (Peinado et al., 2017; Zanconato et al., 2019). The development of metastatic colonies depends on establishing and maintaining a supportive microenvironment, which includes innate and adaptive immune cells and resident stromal cells (Quail and Joyce, 2013; Lambert et al., 2017). In addition, the cellular and extracellular components of the metastatic microenvironment are crucial for metastatic colonization (Lin et al., 2018a; Altorki et al., 2019). Several facts have suggested that lncRNAs have a major influence on the TME (Fatima and Nawaz, 2017; Lin et al., 2018a; Sun et al., 2018a). Tumor cells after detachment from their primary site and traveling *via* the lymphatic and circulatory systems possess the potential to resist cell death, i.e., “anoikis”. The anoikis resistance (AR) is a cornerstone step for metastasis, promoting secondary tumor formation in distal organs (Simpson et al., 2008). Extensive studies have shown that lncRNAs can regulate the AR of cancer cells through modulating pathways, apoptosis-associated proteins, and other molecules (Lee et al., 2021). Primary tumors can influence the microenvironment of distant organs to create a pre-metastatic niche, which can facilitate the spread and colonization of cancer cells. This is achieved through changes in various factors such as inflammation, lymph angiogenesis, immunosuppression, angiogenesis, vascular permeability, and organotropism. These changes create a supportive environment for cancer cells to thrive and establish secondary tumors (Guo et al., 2019), which are important for metastasis development (Nogués et al., 2018). It is well established that lncRNAs play a crucial role in the regulation of tumor progression and can contribute to the development of metastasis. Imbalances of lncRNAs have been shown to be involved in the release of exosomes, alteration of remote tumor cells, creation of a pre-metastatic niche, and survival of disseminated tumor cells (Kim et al., 2016; Liu et al., 2019a). Many studies have also demonstrated that lncRNAs can act as mediators of both intra- and extracellular signaling pathways that drive metastasis, making the targeting of lncRNAs a potential strategy for treating metastatic cancer.

**FIGURE 1:**
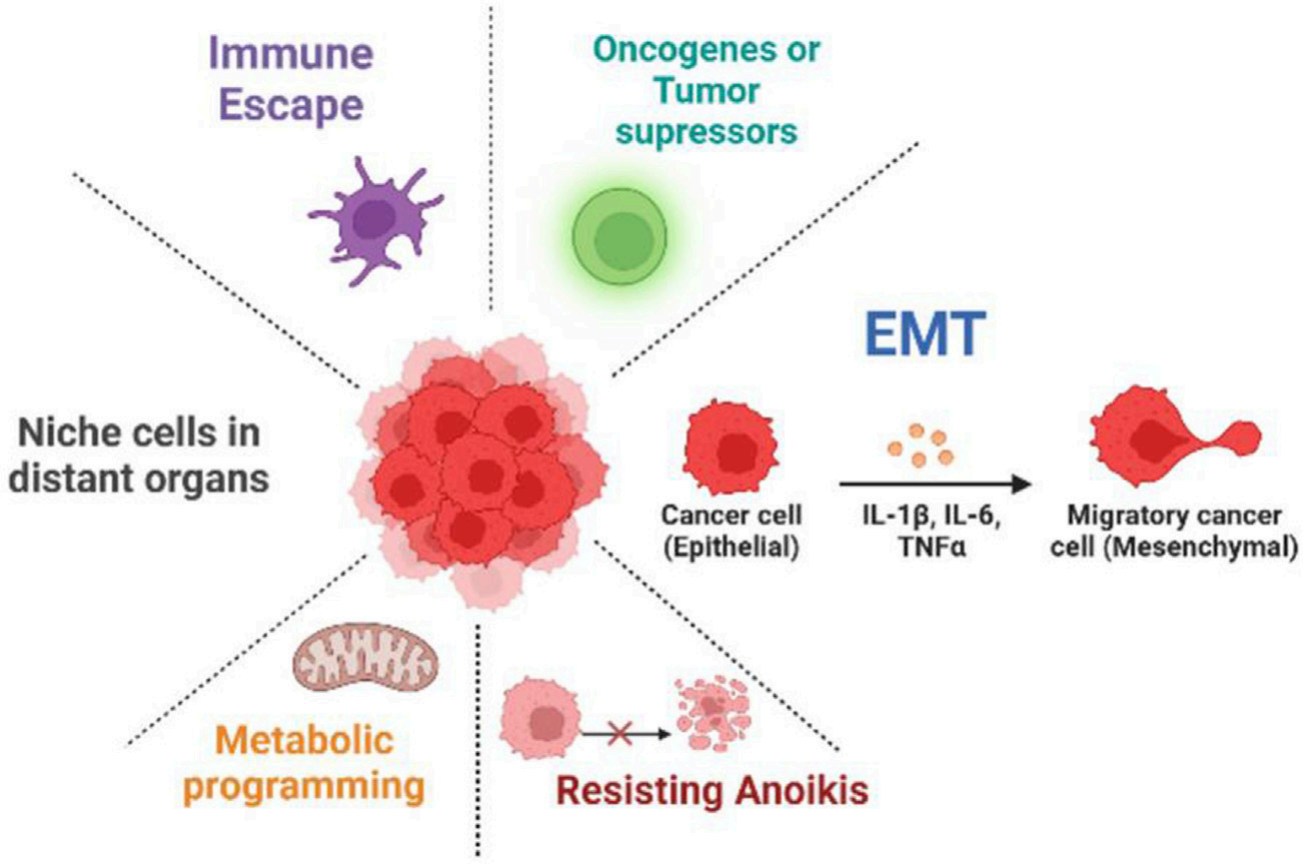
Mechanism of long non-coding RNAs (lncRNAs) in cancer metastasis. lncRNAs play a role in cancer development and growth by regulating various processes such as metabolic reprogramming, anoikis resistance, EMT, metastatic niche, immune escape, and oncogenes or tumor suppressors.

In this review article, the functions of lncRNAs in the development of metastasis will be discussed, such as their involvement in underlying molecular mechanisms, metabolic reprogramming, EMT programming, TME influence, AR, and interaction with pre-metastatic niche formation (Figure 1). The diagnostic, prognostic, and therapeutic potential of lncRNAs in cancers will also be examined. Further research in this field is necessary to gain a comprehensive understanding of the molecular basis of lncRNA-modulated cancer metastasis and develop new diagnostic and therapeutic approaches for patients with metastatic disease. Furthermore, we represented areas for future research in this rapidly developing field.

## Long non-coding RNAs and metabolic reprogramming during cancer metastasis

Otto Warburg in the 1920s identified cancer cells favoring glycolysis over the considerably more effective oxidative phosphorylation (OXPHOS) to generate energy (Warburg, 1956; Vander Heiden et al., 2009), resulting in considerably increased secretion of lactate and glucose uptake in tumor cells (Dang, 2010). This process is described as the Warburg effect, also known as aerobic glycolysis, and represents a new phase in the research of tumor metabolism. Deletions, gene mutations, and translocations impact cancer cells in various signaling pathways and mainly converges at the metabolism level (Cairns et al., 2011; Beloribi-Djefaflia et al., 2016). Cancer cell metabolites not only provide materials for metastasis and for their proliferation but also supply sustaining signals in tumor-specific microenvironments to meet their survival needs (Hanahan and Weinberg, 2011; Alderton, 2014; Weber, 2016). Moreover, cancer cells also perturb the metabolism of distant organs in order to disseminate and ease their growth and implantation (Fong et al., 2015). In turn, metabolic alterations of invaded tissues and organs also affect the survival and growth of carcinoma cells (Mashimo et al., 2014; Loo et al., 2015). Many researchers have recognized that the Warburg effect facilitates inhibition of anoikis (AR) and spread of tumor cells (Lu et al., 2015; Lu, 2019). This ability of tumor cells to resist anoikis (AR) and metastatic spread is provided by aerobic glycolysis, which accelerates glucose consumption, decreases the production of reactive oxygen species (ROS), and enhances the anti-oxidant capacity of tumor cells (Lu et al., 2015). However, despite the Warburg effect, oxidative metabolism is still a significant source of ATP in some cancers (Guppy et al., 2002; Marin-Valencia et al., 2012). Oxidative metabolism in mitochondria can generate reactive ROS that include hydroxyl radicals (HO^−^), hydrogen peroxide (H_2_O_2_), and superoxide (O^**−**2^) (Katritsis et al., 1991). These ROS have been shown to play a role in promoting tumor spread by preventing the death of detached cancer cells (anoikis) (Moloney and Cotter, 2018). A growing body of evidence has shown a connection between ncRNAs, particularly miRNA and lncRNA, and metabolic alterations in cancer (Singh et al., 2012; Sun et al., 2018b). lncRNAs regulate the signaling pathways in key metabolic reprogramming and promote cancer progression, tumorigenesis, and metastasis. Despite reduced glucose levels in solid tumors, enhanced glycolysis produces metabolic intermediate that serves as crucial synthetic components for developing tumors (DeBerardinis et al., 2008) and promotes metastasis (Warburg, 1956; Vander Heiden et al., 2009). More specifically, glycolysis promotes acidic and hypoxic TME associated with the protonation of significant pH-sensitive protein residues (Magalhaes et al., 2011; Choi et al., 2013) and regulates the function and sub-cellular localization of cytoskeleton proteins important for invasion and immune escape (Huber et al., 2017; Rohani et al., 2019). For example, in tumor tissue hexokinases (HKs), the essential glycolytic enzyme has been shown to keep a rapid rate of glycolysis and help cancer cell metastasis (Zhang et al., 2017a). More specifically, HK2 by regulating the matrix metalloproteinase 9 (MMP-9) expression, non-processed pseudogene (NANOG), and SRY box transcription factor (SOX)-9 facilitates the metastasis of ovarian cancer (OVC) cells (Siu et al., 2019). Interestingly, knocking down (KD) of ncRNA-TUG1 and suppressing the miR-455-3p expression leads to decreased activity of adenosine monophosphate–activated protein kinase subunit b2 (AMPKb2), which in turn affects HK2 and reduces the migration and invasion of hepatocellular carcinoma (HCC) cells (Lin et al., 2018b). In gallbladder cancer (GBC) tissues, increased expression of lncRNA PVT1 was negatively correlated with the overall survival (OS) of patients (Chen et al., 2019). Furthermore, KD of lncRNA PVT1 in GBC cells decreased HK2 expression, inhibited glycolysis, and decreased metastases *via* competitive interaction with endogenous miR-143 (Chen et al., 2019).

**FIGURE 2:**
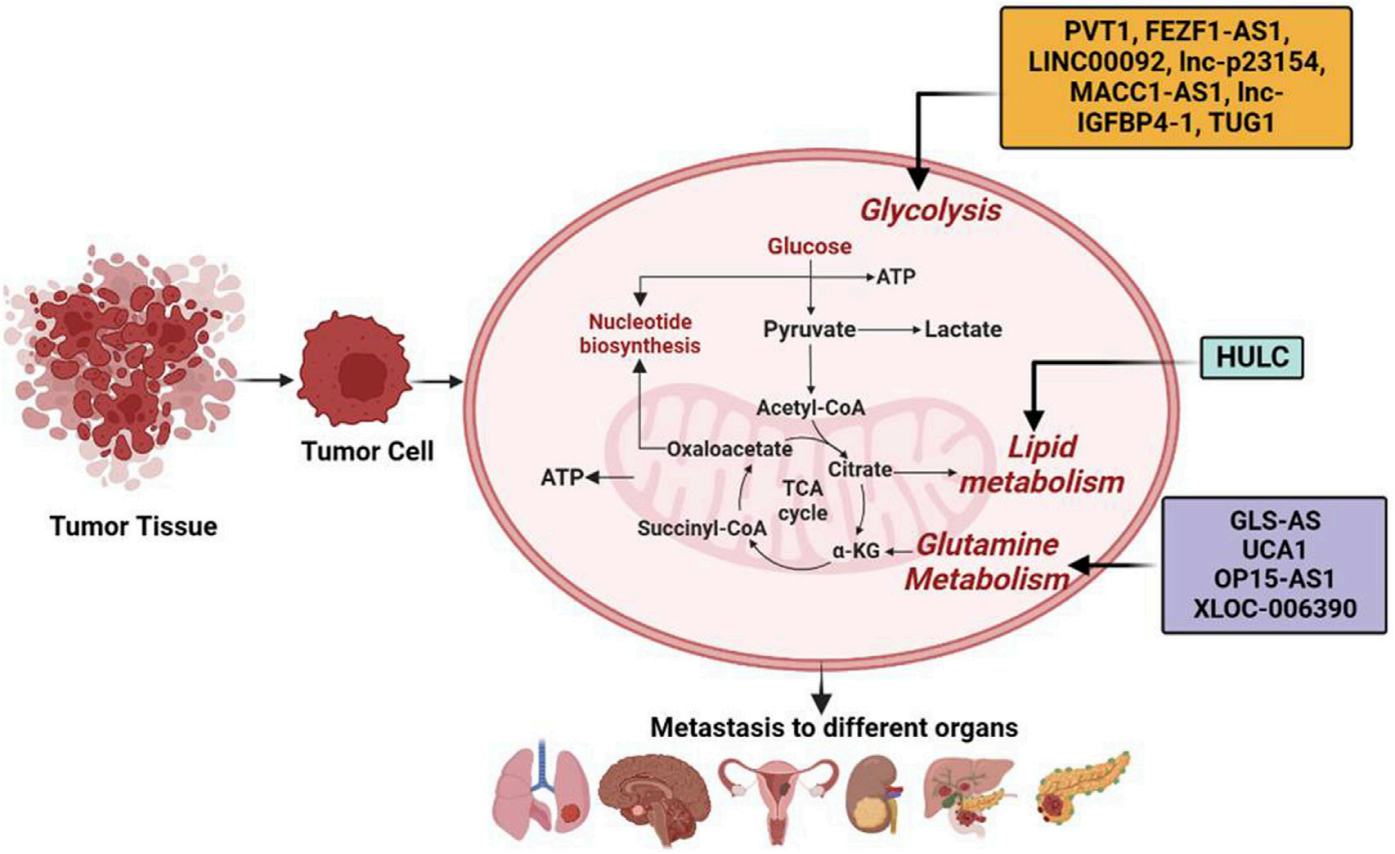
Long non-coding RNAs (lncRNAs) by modulating metabolic reprogramming participate in tumor metastasis. lncRNAs such as PVT1, FEZF1-AS1, LINC00092, LINC00963, MACC1-AS1, HULC, GLS-AS, lnc-p23154, lnc-IGFBP4-1, TUG1, UCA1, OP15-AS1, and XLOC-006390 have been shown to modulate metabolic pathways such as glycolysis, lipid metabolism, and glutamine metabolism, leading to tumor metastasis in different organs.

The lncRNA SAMMSON and MALAT1 have been shown to play roles in the development of melanoma (Leucci et al., 2016) and HCC (Malakar et al., 2019), respectively. SAMMSON interacts with protein p32 to increase its pro-oncogenic function and enhance mitochondrial targeting (Leucci et al., 2016). MALAT1 contributes to HCC by modulating glucose metabolism and enhancing glycolysis while inhibiting gluconeogenesis. It does so by activating the mTORC1-4EBP1 axis, leading to increased translation of the transcription factor TCF7L2 (Malakar et al., 2019). Similarly, lncRNA MACC1-AS1 overexpression is associated with metastasis of gastric cancer (GC) cells to the lungs. The underlying mechanisms include the activation of AMPK/Lin28 pathway–mediated increased glycolysis and anti-oxidative abilities with strong metabolic plasticity (Zhao et al., 2018). lncRNA MACC1-AS1 in pancreatic cancer (PC) is also upregulated and associated with bad prognosis (Qi et al., 2019), its KD prevents the metastasis of PC cells by promoting the expression of paired-box gene 8 (PAX8), which is essential in activating NOTCH 1 signaling and enhancing cell aerobic glycolysis (Qi et al., 2019). An alternate spliced form of PK is pyruvate kinase M2 (PKM2), which is overexpressed in different types of cancerous cells. PKM2 regulates the final rate-limiting step of glycolysis and determines the efficiency of lactic acid production and glucose utilization (Chaneton and Gottlieb, 2012). Histone deacetylase 3 is recruited by direct interaction of transforming growth factor β (TGF-β)–induced factor homeobox 2 (TGIF2) in the nucleus with PKM2 and subsequent deacetylation to the E-cadherin promoter, thus suppressing E-cadherin transcription in colon cancer cells and promoting EMT (Hamabe et al., 2014). lncRNA FEZ finger zinc 1 antisense 1 (lncRNA FEZF1-AS1) is typically overexpressed in colorectal cancers (CRC) that leads to cell metastasis. Mechanistically, PKM2 is stabilized by the binding of FEZF1-AS1 to it, increases aerobic glycolysis, and stimulates the signal transducer and activator of transcription 3 (STAT3) signaling pathway (Bian et al., 2018). Transmembrane glycoprotein known as glucose transporter (GLUT) is an essential factor in the uptake of glucose by cancer cells. A high expression of GLUT1 increases glucose absorption, promoting glycolysis and cancer cell metastasis (Nagarajan et al., 2017). The gene expression of GLUT1 is correlated with the activity of MMP-2 and invasiveness in PC (Ito et al., 2004). lnc-p23154 regulates glycolysis by inhibiting the transcription of miR-378a-3p, resulting in increased expression of GLUT1, which contributes to the development of oral squamous cell carcinoma (OSCC) metastasis (Wang et al., 2018a). Lactate dehydrogenase A (LDHA) is the rate-limiting enzyme in the metabolic pathway that converts glucose into lactate and pyruvate. LDHA is an important enzyme that regulates the balance between energy production and energy utilization in the cell, and its activity has been shown to be altered in various diseases such as cancer. The progression of breast cancer (BC) metastasis is positively correlated with the phosphorylation of enzyme LDHA at Y10 (Jin et al., 2017). LDHA induces EMT and facilitates the metastasis of lung adenocarcinoma cells (LADCs) (Hou et al., 2019). In addition, many other kinases and glycolytic enzymes involved in glycolysis are also regulated by lncRNAs. For example, LINC00092 suppresses the enzyme 6-phosphofructo-2-kinase/fructose-2,6-biphosphatase 2 (PFKFB2), which is involved in the synthesis and breakdown of fructose-2,6-bisphosphate (Zhao et al., 2017). It is important to mention that chemokine-CXCL14 (C-X-C motif ligand 14) is overexpressed in cancer-associated fibroblasts (CAFs) in OVC and induces LINC00092 expression. For cancerous cells, a non-essential amino acid glutamine has vital biological importance with enhanced glutamine uptake and catabolism. Glutamate dehydrogenase (GDH) catalyzes the conversion of glutamine into glutamate, which is triggered by an enzyme glutaminase (GLS), and results in the production of α-ketoglutarate, an intermediary for the TCA cycle and an important source of energy. Glutamine has been demonstrated to increase hypoxia-inducible factor-1 (HIF1) expression and also increase the pro-autophagic function of BNIP3 (Bcl2/adenovirus E1B interacting protein 3), thus encouraging melanoma cell dissemination (Vara-Perez et al., 2019). Fumarate, an intermediate product of glutamine metabolism, induce metastasis by activating glutathione peroxidase, and reducing the ROS levels (Jin et al., 2015). The c-Myc protein is a transcription factor that plays a role in the regulation of gene expression and cell growth. One mechanism by which c-Myc contributes to cancer is by upregulating the expression of miR-23b, which in turn suppresses the expression of proline oxidase and leads to an increase in glutamine catabolism to promote cell proliferation and contribute to the development of cancer (Liu et al., 2012). Interestingly, lncRNA GLS-AS has been shown to regulate glutaminase and c-Myc feedback loop, thus contributing to the metastasis of PC cells (Deng et al., 2019). In bladder cancer (BDC), lncRNA UCA1 and GLS2 are positively correlated. UCA1 has a positive effect on human BDC cells by reducing ROS production and promoting mitochondrial glutaminolysis, as well as having sponge effects on miR-16. The binding of miR-16 “seed region” to the 3′-UTR of GLS2 mRNA regulates GLS2 expression (Li et al., 2015a). Similarly, lncRNA OIP5-AS1 is upregulated in melanoma by sponge miR-217, which leads to increased GLS expression and promotion of glutamine catabolism and melanoma growth (Luan et al., 2019). For maintaining cellular energy homeostasis, 5′ adenosine monophosphate–activated protein kinase (AMPK) is an essential detector. AMPK phosphorylation of sterol regulatory element binding protein-1 (SREBP1) and acetyl-CoA carboxylase (ACC1) restricts the production of cholesterol, fatty acids, and triglycerides while promoting the uptake of fatty acids. Moreover, AMPK also induces glycolysis by activating the phosphorylation of glycogen phosphorylase and PFKFB3 (Hardie et al., 2012). However, reduced AMPK activity is associated with increased fatty acid synthesis and cancer cell growth (Kim et al., 2011). Tumor suppressor liver kinase B1 (LKB1), an upstream kinase of AMPK, phosphorylates and activates AMPK during ATP-depleted conditions (Hezel and Bardeesy, 2008). Under the TME, activation of the LKB1-AMPK signaling pathway inhibits autophagy *via* miR-7 upregulation and decreases intracellular glucose supply, coupled with decreased proliferation and metastasis of PC cells (Gu et al., 2017). In HCC cells, taurine-upregulated gene 1 (TUG1) through the miR-455-3p/AMPKb2 axis upregulates the HK2 expression, promotes glycolysis, and induces metastasis (Lin et al., 2018b). Similar to AMPK, the hyperactivation of the PI3K/AKT/mTOR signaling pathway promotes cancer proliferation, growth, invasion, and metastasis (Jeong et al., 2014; Hua et al., 2018). LINC00963 promotes metastasis in non–small-cell lung cancer (NSCLC) by stimulating the AKT/mTOR pathway and by inhibiting the ubiquitination of phosphoglycerate kinase 1 (PGK1) (Yu et al., 2017). Likewise, the high expression of the long non-coding RNA MACC1-AS1 has been linked to the spread of GC cells to the lungs. This is due to the activation of the AMPK/Lin28 pathway, which enhances glycolysis and anti-oxidative capacity, resulting in improved metabolic adaptability (Zhao et al., 2018). In PC, lncRNA MACC1-AS1 is overexpressed and associated with poor prognosis (Qi et al., 2019). KD MACC1-AS1 has been shown to inhibit metastasis of PC cells by increasing the expression of paired-box gene 8 (PAX8), which is crucial for activating NOTCH 1 signaling and improving aerobic glycolysis of the cells (Qi et al., 2019). Furthermore, in HCC, lncRNA HULC is highly overexpressed and acts as an oncogene. It modulates lipid metabolism by involving miR-9, PPARA, and ACSL1 in a signaling pathway, strengthened by a feed-forward mechanism involving cholesterol and RXRA to drive HULC signaling (Cui et al., 2015) (Figure 2, Table 1).

**TABLE 1:** lncRNAs involved in regulating tumor metabolism.

lncRNA	Target	Action	Tumor type	Ref.
Glucose metabolism				
TUG1	miR-455-3P	Up	Hepatocellular carcinoma	Lin et al. (2018b)
PVT1	miR-143	Up	Gallbladder cancer	Chen et al. (2019)
lncRNA FEZF1-AS1	PKM2	Up	Colorectal cancer	Nagarajan et al. (2017)
lnc-p23154	miR-378a-3p	Up	Oral squamous cell carcinoma	Wang et al. (2018a)
SAMMSON	P32	Interact	Melanoma	Leucci et al. (2016)
MALAT1	TCF7L2	Up	Hepatocellular carcinoma	Malakar et al. (2019)
lncRNA MACC1-AS1	AMPK/Lin28	Up	Gastric cancer	Zhao et al. (2018)
lncRNA MACC1-AS1	PAX8	Up	Pancreatic cancer	Qi et al. (2019)
LINC00092	PFKFB2	Up	Ovarian cancer	Zhao et al. (2017)
LINC00963	PGK1	Up	Non–small-cell lung tumor	Yu et al. (2017)
Lipid metabolism				
HULC	miR-9	Up	Hepatocellular carcinoma	Cui et al. (2015)
Glutamine metabolism				
GLS-AS	GLS	Down	Pancreatic cancer	Deng et al. (2019)
OIP5-AS1	miR-217	Up	Melanoma	Luan et al. (2019)
UCA1	miR-16	Up	Bladder cancer	Li et al. (2015a)

Up/down indicates lncRNA upregulating or downregulating the expression of target genes. PKM2, pyruvate kinase isozymes M2; GLS, glutaminase; PFKFB, 2,6-phosphofructo-2-kinase/fructose-2,6-biphosphatase; TCF7L2, transcription factor 7-like 2; LDH, lactate dehydrogenase.

## lncRNAs promote epithelial–mesenchymal transition during cancer metastasis

EMT is an essential part in the cell dissemination process that enables cancer cells to leave the original site and migrate to distant regions (Nieto et al., 2016; Brabletz et al., 2018). EMT can be activated by numerous intracellular signaling pathways that are activated once the ligands that originate from the stroma bind to the appropriate receptors that are expressed in neoplastic cells (Jiang et al., 2017a; Dongre and Weinberg, 2019). Recent research have demonstrated that lncRNAs are significant EMT modulators in tumor metastasis (Guo et al., 2014; Jia et al., 2016; Heery et al., 2017). Accumulated evidence has demonstrated that NOTCH, TGF-β/SMAD, PI3K/AKT, Wnt/β-catenin, JAK/STAT, and MEK/ERK are responsible for inducing the EMT-activating transcription factors (EMT-TFs) expression, particularly SNAIL, TWIST, and ZEB, which simultaneously prevent the epithelial state–related gene expression and enhance the expression of genes linked to the mesenchymal state (Xu et al., 2009; Bray, 2016; Nieto et al., 2016; Yang et al., 2017; Browning et al., 2018; Cevenini et al., 2018; Tauriello et al., 2018; Dongre and Weinberg, 2019; Su et al., 2020). lncRNAs have been found to play a significant role in regulating the TGF-β signaling pathway in cancers (Wang et al., 2016). LINC00978 (also known as AK001796 and MIR4435-2HG) promotes EMT in GC (Fu et al., 2018). KD of LINC00978 inactivates SMAD2 and reduces TGF-β expression, leading to decreased expression of MMP-9, SNAIL2, and TWIST1 genes (Fu et al., 2018). Likewise, lncRNA TUG1 induces EMT in PC by controlling the TGF-β signaling pathway. It decreases SMAD4 expression and increases TGF-β and TGF-β receptor expressions, leading to the induction of EMT (Qin and Zhao, 2017). Similarly, lncRNA actin filament–associated protein-1 antisense RNA1 (lncRNA AFAP1-AS1) modulates the expression of several EMT-related genes (*SLUG*, *SNAIL1*, *VIM*, *CADN*, *ZEB*, and *TWIST*) in tongue squamous cell carcinoma (TSCC) by controlling the Wnt/β-catenin signaling pathway (Wang et al., 2018b). Additionally, the polycomb repressive complex 2 (PRC2) and lncRNA HOTAIR regulate WIF-1 expression by increasing H3K27 methylation in the promoter region, activating the Wnt/β-catenin signaling pathway in esophageal squamous cell carcinoma (ESCC). This activation is confirmed by the overexpression of downstream genes such as MMP-13, ZEB1, and SNAIL (Ge et al., 2013). NOTCH is a well-known oncogene involved in tumor cell proliferation, AR, and EMT in many cancers (Shao et al., 2015; Wieland et al., 2017; Jackstadt et al., 2019). lncRNAs have been shown to directly bind to the NOTCH pathway’s core components and regulate many EMT-TFs. For example, lncRNA hepatocyte nuclear factor-1 alpha antisense RNA 1 (lncRNA HNF1A-AS1) promotes EMT in oral squamous cell carcinoma (OSCC) by upregulating the NOTCH 1 and Hes-1 expression (Liu et al., 2019b). In addition, lncRNAs can also act as competitive endogenous RNAs (ceRNAs), indirectly influencing NOTCH signaling by working with the TGF-β pathway to advance EMT. By sponging miR-124, the lncRNA urothelial cancer–associated 1 (lncRNA UCA1) regulates the TGF-β1 expression to upregulate JAG1 and activate NOTCH signaling in tongue cancer (TC) cells (Zhang et al., 2019b).

**FIGURE 3:**
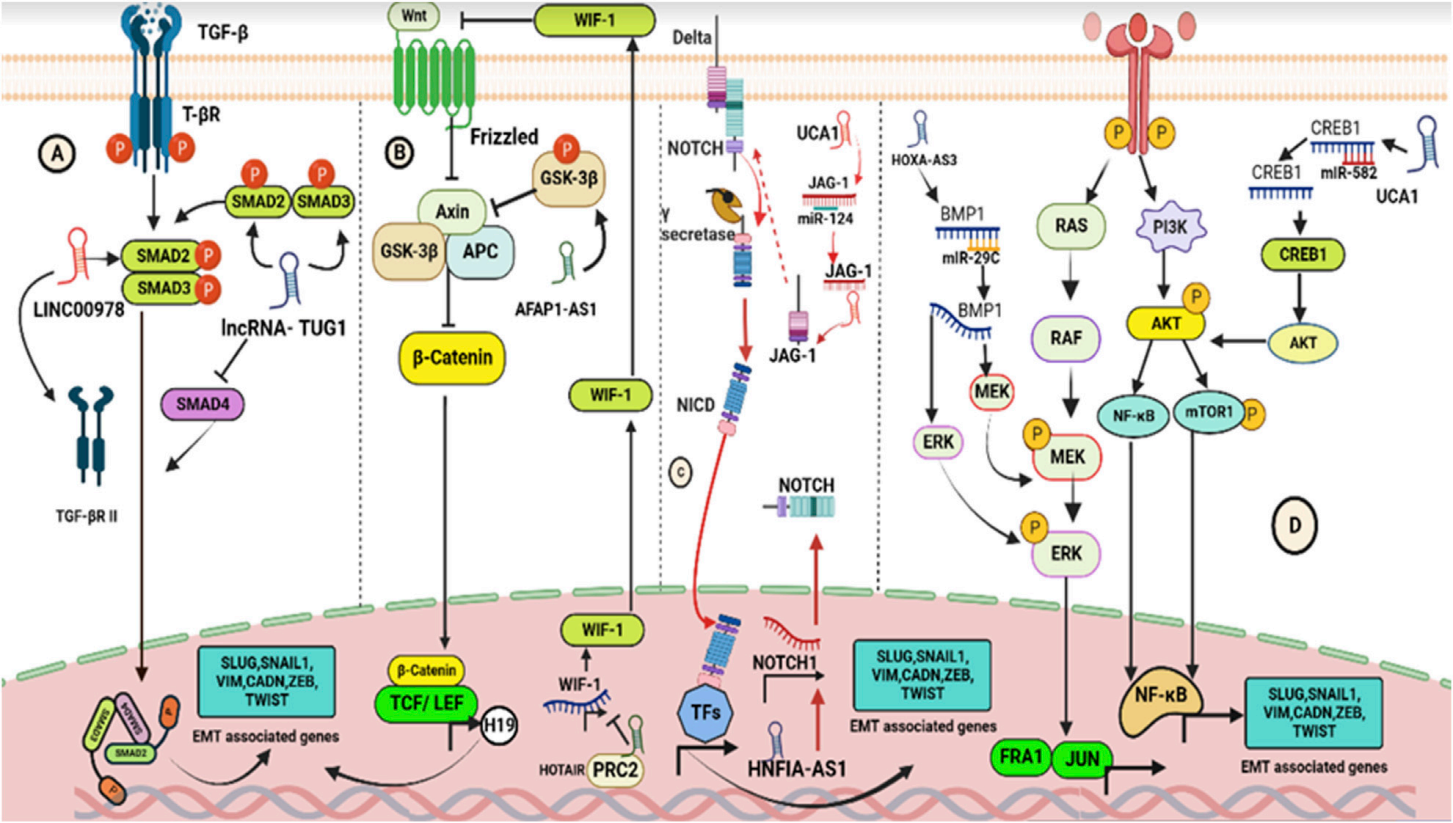
Schematics showing pathways modulated by lncRNAs to induce EMT. **(A)** Interaction of TGF-β ligands with T-βR triggers the canonical TGF-pathway, leading to the formation of trimeric SMAD complexes (SMAD2–SMAD3–SMAD4) which act as transcription factors in the nucleus to regulate the expression of EMT-associated genes. lncRNAs, such as LINC00978 and TUG1, can modulate the TGF-β/SMAD signaling transduction by affecting the expression and phosphorylation of SMAD2/3 and SMAD4, respectively. **(B)** Interaction of Wnt ligands with frizzled receptors triggers the canonical Wnt pathway, leading to the release of β-catenin which binds with LEF and TCF to promote the expression of EMT-related genes. lncRNAs AFAP1-AS1 and HOTAIR modulate the Wnt/β-catenin pathway by affecting the phosphorylation of GSK3β and methylation of H3K27, respectively. **(C)** Interaction of Jagged/Delta-like ligands with Notch receptors triggers the canonical Notch pathway, leading to the generation of NICD which acts as a transcriptional co-activator in the nucleus. lncRNAs HNF1A-AS1 and UCA1 modulate the Notch pathway by regulating the expression of essential components and acting as ceRNA to regulate Notch signaling indirectly. **(D)** Growth factors activating the MEK-ERK and PI3K-AKT pathways also induce EMT through simultaneous activation of EMT-TFs. lncRNAs, such as UCA1 and HOXA-AS3, can act as ceRNA to regulate the expression of CREB1 and miR-29c, by promoting PIK3/AKT/mTOR pathway, and enhance the phosphorylation of MEK and ERK, respectively.

All these studies conclusively establish that lncRNAs enhance EMT through the upregulation of the canonical Wnt/β-catenin and the activation of the NOTCH pathways. Multiple intercellular signaling pathways, such as PI3K/AKT/mTOR, are activated to induce EMT (Grotegut et al., 2006; Di Domenico and Giordano, 2017), and lncRNAs have been shown to regulate these intercellular signaling pathways in cancer metastasis. Specifically, lncRNA UCA1 positively modulates the PI3K/AKT/mTOR signaling pathway by sponging miR-582 and activating the target protein CAMP responsive element binding protein 1 (CREB1), inducing EMT in osteosarcoma cells (Ma et al., 2019). lncRNA TTN-AS1 activates the PI3K/AKT/mTOR signaling pathway by sponging miR-497 and promoting metastasis of colorectal cancer cells. lncRNA HOXA-AS3 regulates the MEK/ERK signaling pathway by targeting miR-29c and controlling BMP1 expression, which facilitates EMT in HCC (Cui et al., 2019a) (Figure 3). Further research is required to fully understand the molecular mechanisms of lncRNA regulation of signaling pathways in cancer metastasis.

## lncRNAs modulate tumor microenvironment during metastasis

The TME comprises of the extracellular matrix (ECM), basement membrane (BM), tumor-infiltrating immune cells, neuroendocrine cells, endothelial cells, adipose cells, cancer-associated fibroblasts (CAFs), pericytes, cancer stem cells (CSCs), cytokines, and a plethora of signaling molecules that modulate tumor progression (Macha et al., 2020; Bhat et al., 2021a; Bhat et al., 2021b; Nisar et al., 2021b; Mehraj et al., 2021; Neophytou et al., 2021; Bhat et al., 2022). The interactions between tumor cells and their microenvironment are very critical for cell survival, and therefore impact the development and growth of tumors (Gajewski et al., 2013; Bhat et al., 2021a; Bhat et al., 2021b; Nisar et al., 2021b; Bhat et al., 2022). It also plays a crucial role in cancer metastasis by providing the necessary support and signals for tumor survival, growth, and migration to new sites in the body. lncRNAs are essential players in the crosstalk between cancer cells and the TME. Numerous studies have demonstrated that lncRNAs promote the formation of an immunosuppressive tumor immune microenvironment (TIME), which contributes to tumor escape from immune surveillance, promoting metastatic development and therapeutic resistance (Pei et al., 2018; Wei et al., 2019a). Neutrophils are immune cells that can exhibit different polarization states, such as the tumor-promoting N2 type and the anti-tumor N1 type, which are influenced by the TME. N2-type neutrophils promote tumors by increasing angiogenesis, facilitating tumor cell infiltration and ECM reconstruction, inhibiting T-cell activation, and inducing anti-inflammatory M2 macrophages (Guo et al., 2022). KD of LINC01116 in CAFs leads to the generation of tumor-associated neutrophils (TANs), which promote tumor growth through the secretion of cytokines (Wang et al., 2020). The lncRNA Morrbid regulates neutrophil lifespan and apoptosis, making it a potential therapeutic target in the TIME (Coffelt et al., 2016; Kotzin et al., 2016). In OVC cells, the lncRNA HOTTIP enhances immunosuppression by increasing PD-L1 expression in the neutrophils and upregulating IL-6 expression (Shang et al., 2019).

**FIGURE 4:**
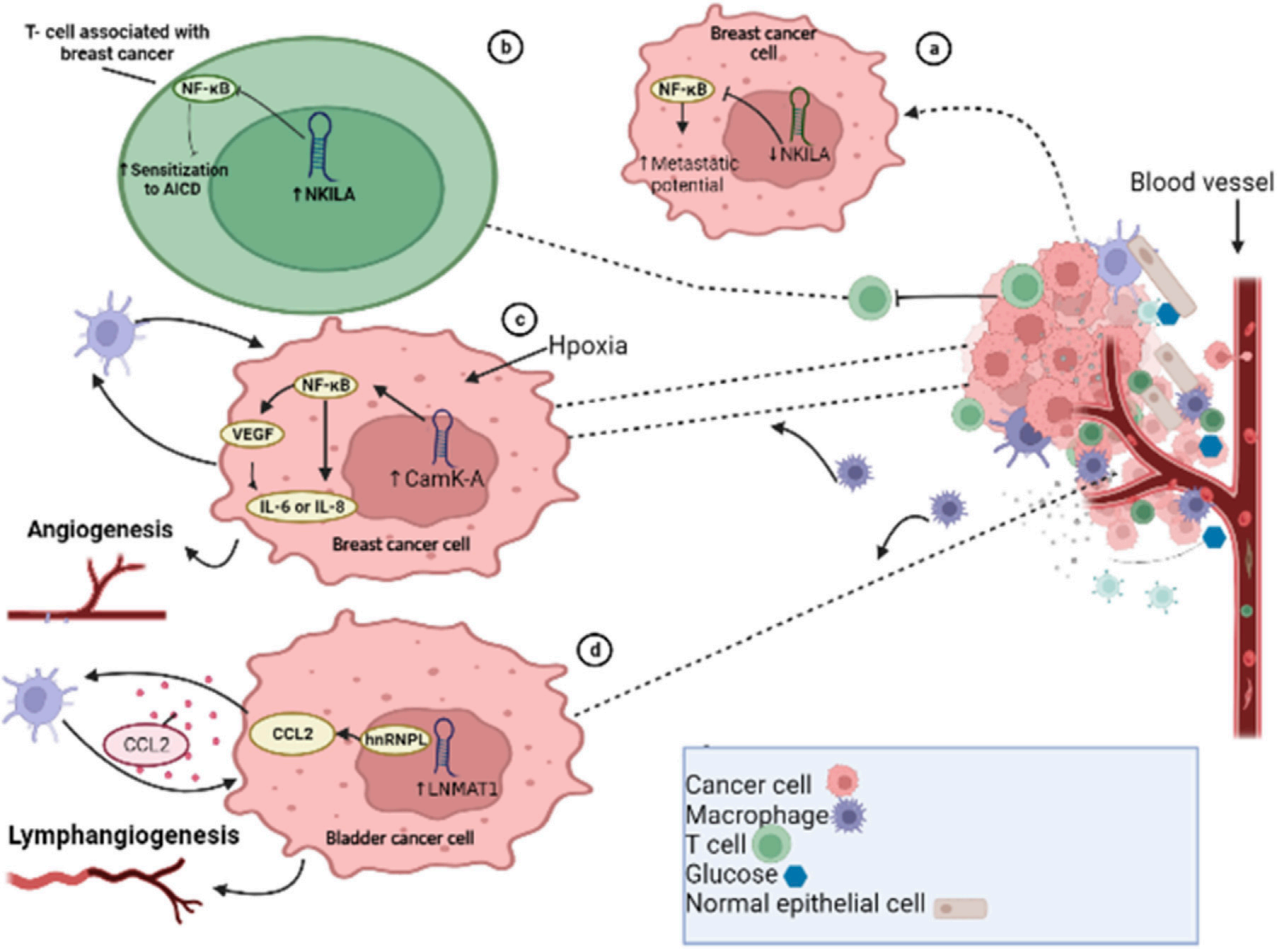
Long non-coding RNAs modulate tumor microenvironment. **(A)** lncRNA NKILA inactivates the nuclear factor-kappa beta (NF-kB) pathway in breast cancer cells. **(B)** Downregulation of lncRNA NKILA in tumor-infiltrating T lymphocytes leads to decreased metastatic potential. **(C)** In BC cells, in response to micro environmental hypoxia, activation of CamK-A triggers the activation of the NF-kB pathway, leading to increased expression of IL6, IL8, and VEGF, and enhances angiogenesis. **(D)** In bladder cancer metastasis, the lncRNA LNMAT1 recruits hnRNPL to the CCL2 promoter, resulting in increased expression of CCL2 through CCL2-dependent macrophage recruitment.

Similar to neutrophils, macrophages are immune cells that play a crucial role in both innate and adaptive immune responses. The two main phenotypes, M1 and M2, have distinct functions in the body. The M1-type macrophage prevents pathogen invasion and destroys tumor cells while M2 macrophages predominantly enhance metastasis, invasion, and tumor development. These specific M2 macrophages are known as tumor-associated macrophages (TAMs) by exerting immunosuppressive and tumor-promoting effects (Ruffell et al., 2012; DeNardo and Ruffell, 2019). M2 macrophages have anti-inflammatory characteristics and facilitate the formation of tumors, by promoting angiogenesis, tumor cell infiltration, tumor cell proliferation, and metastasis, and suppressing immune function and chemotherapy resistance (Murdoch et al., 2008; De Palma and Lewis, 2013; Shu and Cheng, 2020). The expression of lncRNAs in the macrophages also impacts macrophage recruitment, tumor development, and progression, affecting factors such as invasion, metastasis, and vascularization. Calcium (Ca^2+^)-dependent signaling and Ca^2+^ flux play significant roles in the development and progression of tumors. In response to hypoxia-induced Ca^2+^ influx, lncRNA calcium-dependent kinase activation (lncRNA CamK-A) activates NF-κB by degrading IκB to upregulate the expressions of *IL6*, *IL8*, and *VEGF* in BCs, promoting angiogenesis and macrophage recruitment in patient-derived BC xenografts (Sang et al., 2018) (Figure 4). The expression of FOXO1 can be targeted and inhibited by lncRNA ANCR, which promotes M2-macrophage polarization, and thus enhances tumor cell migration and invasion (Xie et al., 2020). TAMs, which have similar effects to M2-macrophages, can facilitate tumor development and angiogenesis through lncRNA RP11-361F15.2, which acts as a ceRNA and sponges miR-30c-5p, activating and binding CPEB4 and enhancing the progression and metastasis of osteosarcoma (Yang et al., 2020). The endothelial cells play a crucial role in supporting blood vessel formation and tumor neovasculature. Tumor-associated endothelial cells display a high expression of TGF-β1 and CD105, and TGF-β1 acts as a chemoattractant for CD105-expressing endothelial cells, promoting angiogenesis (Benetti et al., 2008). *In vitro* studies have shown that KD of taurine upregulated gene 1 (TUG1) leads to remarkable suppression of tumor-induced endothelial cell proliferation, migration, and angiogenesis (Dong et al., 2016). Cytokines such as TNF-α, IFN, and IL-17 are major target molecules in various inflammatory conditions, with targeted therapies already in clinical use (Budhu and Wang, 2006; Rider et al., 2016; Yu et al., 2018). TGF-β plays a complex role in tumor progression (Fabregat and Caballero-Díaz, 2018; Marquardt, 2018), and the lncRNA ATB is induced by TGF-β1 (Yuan et al., 2014). In HCC specimens, lncRNA ATB was found to be overexpressed, and it enhanced EMT and metastasis by increasing the colonization of migrating cells *via* the IL-11/STAT3 signaling pathway. The ECM is produced by stromal cells in the microenvironment and its components, which include laminin, collagens, fibronectin, and proteoglycans, are associated with altering the phenotype and function of HCC cells. ECM production and reorganization can promote tumor cell proliferation and invasion, alter gene expression in different stromal and cancer cell types, and lead to tumor progression (Frantz et al., 2010). Extracellular proteinases, such as matrix metalloproteinases (MMPs), mediate many of the changes in the TME during tumor progression. In HCC, the amplification of the lncRNA ZFAS1 gene is positively correlated with hepatic invasion and metastasis through modulation of the miR-150/ZEB1/MMP-14/MMP-16 cascade (Li et al., 2015b). Cancer is a heterogeneous population of cells, and CSCs are responsible for metastasis and resistance to traditional therapies (Chiba et al., 2016; Xiao et al., 2017). CSCs or TICs have been identified in multiple cancer types, such as HCC, and are proposed as critical promotors of tumor initiation, development, metastasis, and recurrence. The upregulation of lncRNA UCA1 in liver CSCs plays a critical role in governing their growth and differentiation through regulation of multiple pathways (Gui et al., 2015; Pu et al., 2015).

T cells play a crucial role in both cancer development and immune responses. lncRNAs play a role in T-cell activation, differentiation, development, function, and cancer immunology. T-cell activation by foreign antigens triggers immune responses that are controlled by activation-induced cell death (AICD) of T lymphocytes. Cancer cells use AICD to evade the immune system. lncRNA has been shown to be involved in AICD of T lymphocytes, and NF-KappaB Interacting LncRNA (lncRNA NKILA) has been found to modulate T-cell sensitivity to AICD by inhibiting NF-kB activity (Huang et al., 2018). KD of NKILA has been shown to suppress BC progression and enhance the infiltration of cytotoxic T lymphocytes (CTLs) (Huang et al., 2018). A previous study found that in BC cells, cytoplasmic lncRNA that directly inhibits the NF-kB complex leads to increased metastatic potential when downregulated (Liu et al., 2021) (Figure 4). In the TME, T cells have the ability to activate and differentiate into regulatory T cells (Tregs). Tregs play a crucial role in the regulation of tumor immunity by inhibiting the anti-tumor immunity of immune cells such as NK cells, CD8^+^ T cells, and DCs (Wang et al., 2011). lncRNAs have been found to influence the function of Tregs in the TME and inhibit immune surveillance (Jiang et al., 2017b). Some lncRNAs, such as SNHG1, lnc-epidermal growth factor receptor (lnc-EGFR), Flicr, and Flatr, have been documented to modulate Tregs (Jiang et al., 2017b; Zemmour et al., 2017; Brajic et al., 2018). lncRNA LNMAT1 has also been shown to induce metastasis of the lymphatic system in BDC patients by recruiting macrophages and enhancing H3K4 tri-methylation and CCL2 expression by recruiting hnRNPL (heterogeneous nuclear ribonucleoprotein L) to its promoter (Chen et al., 2018) (Figure 4).

CAFs are one of the core components of the TME, which have a significant role in reshaping the ECM structure. This can lead to collagen tracking and promote the migration of cancer cells (Guo et al., 2022). lncRNAs have also been found to be key drivers of CAF-mediated metastatic progression. For example, LINC00092 interacts with a glycolytic enzyme, 6-phosphofructo-2-kinase/fructose-2,6-biphosphatase 2 (PFKFB2), to regulate the level of glycolysis and support CAF activity, thereby promoting OVC metastasis (Zhao et al., 2017). Additionally, exosomal LINC00659 transferred from CAFs can increase the expression of ANXA2 by binding with miR-342-3p, thereby enhancing EMT(, and migration and proliferation of CRC cells (Zhou et al., 2021).

The TME also plays a crucial role in immune escape, as tumor-related immunosuppressive factors can contribute to the ability of cancerous cells to resist the body’s immune surveillance (Ott et al., 2013; Postow et al., 2015). Immune checkpoint inhibitors (ICIs), such as monoclonal antibodies, are designed to block the communication between immune cells and tumor cells and are used to enhance the function of the immune system in the fight against cancer. Tumor-associated immune checkpoint molecules include LAG3, TIM-3, PD-1, and CTLA4, among others (Tumeh et al., 2014). PD-L1 on tumor cells can interact with PD-1 on the surface of relevant lymphocytes, leading to the production of cytokines and lymphocyte apoptosis, thereby allowing tumor cells to evade immune surveillance (Tumeh et al., 2014). lncRNAs have been found to play a crucial role in the regulation of tumor immunity and the development of drug resistance. Some lncRNAs have been shown to promote an immunosuppressive microenvironment that allows tumors to escape the immune system and become resistant to drugs. For example, lncRNA MALAT1 indirectly increases the expression of the immune checkpoint protein PD-L1 through miR-200a-3 and miR-195 binding (Wei et al., 2019b; Wang et al., 2019), while the lncRNA NKX2-1-AS1 has been shown to decrease the PD-L1 expression (Kathuria et al., 2018). Immunotherapy, particularly using PD-1/PD-L1 inhibitors, has made significant advancements in the treatment of solid tumors (Kato et al., 2019; Motzer et al., 2019). However, the modulation of lncRNAs can play a key role in determining the resistance to these ICI therapies. Therefore, it is essential to understand the regulatory functions of lncRNAs in the TME in order to improve the effectiveness of immunotherapy and overcome resistance to these treatments. In conclusion, lncRNAs play a crucial role in regulating various aspects of tumorigenesis, angiogenesis, immunosuppression, and tumor cell progression. A deeper understanding of the regulation of lncRNAs in the TME is necessary for advancing the treatment of metastatic tumors.

## lncRNA regulates anoikis resistance during metastasis

AR is a cellular function that plays a critical role in the spread of cancer by allowing cancer cells to resist death when they detach from the ECM (Sakamoto and Kyprianou, 2010; Tajbakhsh et al., 2019; Lee et al., 2021), mainly during metastasis (Paoli et al., 2013). This capability enables cancer cells to dissociate from the primary tumor site and invade distant areas, establishing a metastatic lesion (Simpson et al., 2008; Sakamoto and Kyprianou, 2010). Therefore, AR is considered a crucial process for tumor cell metastasis and has been the target of research for developing new cancer therapies (Coates et al., 2010; Sakamoto and Kyprianou, 2010). Multiple factors and mechanisms have been linked to AR in cancer cells, such as changes in integrin expression, growth factors, oxidative stress, autophagy, EMT, metabolic alterations, and signaling pathways (Paoli et al., 2013; Adeshakin et al., 2021). Additionally, various ncRNAs, particularly lncRNAs, have been associated with AR in several types of cancer (Lee et al., 2021). One specific lncRNA, HOX transcript antisense intergenic RNA (HOTAIR), has been shown to be upregulated in many cancers and linked to metastasis, aggressiveness, and poor patient prognosis (Liu et al., 2013; Tan et al., 2018). While lncRNA HOTAIR increased the AR of OVC cells by recruiting EZH2 and prompting H3K27 methylation (Dai et al., 2021), its KD decreased the ability of AR, migration, invasion, and spheroid formation (Dai et al., 2021). HOTAIR also facilitates AR in GC (Okugawa et al., 2014) and HCC cells through c-Met signaling (Topel et al., 2020). ANRIL, for example, is positively correlated with glioma and modulates caspase-3/8/9 and AKT signaling pathways by sponging miR-203a (Dai et al., 2018). APOC1P1-3 suppresses early apoptosis and promotes AR by reducing activated caspase 3, 8, 9, and PARP through the specific sponge of target miRNA-188-3p (Lu et al., 2021). lncRNA H19 imprints maternally expressed transcripts and promotes EMT and metastasis in various cancers (Yoshimura et al., 2018), and the downregulation of lncRNA H19 decreases liver and lung metastases in PC cells (Yoshimura et al., 2018). lncRNA H19 also activates the Wnt signaling pathway by sponging miR-29-3b and stimulates the onset of EMT in CRC (Ding et al., 2018). Telomerase reverse transcriptase (TERT), in addition to potentiating cancer stemness, metastasis, and telomere length maintenance (Hannen and Bartsch, 2018), also promotes anchorage-independent growth of cancer cells. lncRNA FOXD2-AS1, by sponging miR-7, promotes anchorage-independent growth of thyroid cancer (THC) cells by targeting TERT (Fleisig and Wong, 2012). lncRNA FOXD2 adjacent opposite strand RNA 1 (lncRNA FOXD2-AS1) has been shown to promote the anchorage-independent growth of THC cells by sponging miR-7 and targeting TERT (Cui et al., 2019b). The inactivation of the Hippo signaling pathway is associated with tumor progression and metastasis in many cancers (Calses et al., 2019; Dey et al., 2020). lncRNA MAPK8IP1P2, by sponging miR-146b-3p, activates the Hippo pathway and inhibits anchorage-independent growth and lymphatic metastasis of thyroid cancer *in vitro* and *in vivo* (Calses et al., 2019; Liu et al., 2020b; Dey et al., 2020). Analysis of the Cancer Genome Atlas (TCGA) database has revealed that lncRNA NEAT1 is dysregulated in several cancers (Li et al., 2018a) and correlated with lymph node metastasis in cervical cancer (CC) (Shen et al., 2020). Increased lncRNA MALAT1 expression has also been associated with advanced tumor stage, recurrence, and reduced survival in OVC (Gordon et al., 2019). In addition, KD of lncRNA MALAT1 expression in anoikis-resistant OVC cells induced apoptosis by modulating RBFOX2-mediated alternative splicing of KIF1B (pro-apoptotic isoform) (Gordon et al., 2019). Furthermore, lncRNA NEAT1 promotes the metastatic potential of endometrial cancer (EndC) by sponging anti-metastatic miR-361 (Dong et al., 2019). lncRNA VAL induces AR by directly abrogating Trim16-dependent vimentin poly-ubiquitination and degradation (Tian et al., 2020). LINC00958 is upregulated in bladder tumor samples (BDC) compared with normal samples, and KD attenuated an AR of BDC cells (Seitz et al., 2017). miR-7 regulates p65 subunit of NF-kB (known as RELA proto-oncogene, NF-kB subunit (RELA), and KLF4 expression to control invasion, angiogenesis, progression, and metastasis (Okuda et al., 2013; Cui et al., 2017; Li et al., 2020b). These functions of miR-7 in breast cancer (BC) can be diminished by the expression of lncRNA TINCR, which is enhanced by the Sp1 transcription factor. Silencing of lncRNA TINCR leads to a reduction in the anchorage-independent growth, migration, invasion, cell survival, and *in vivo* growth of BC cells (Liu et al., 2018) (Table 2). Further research into the mechanisms of AR and its association with lncRNAs may lead to the development of new treatments for cancer.

**TABLE 2:** List of lncRNAs regulating anoikis resistance in various cancers.

LncRNA	Type of cancer	Target/axis	Ref
lncRNA HOTAIR	Ovarian cancer	EZH2/H3K27	Dai et al. (2021)
lncRNA HOTAIR	Hepatocellular carcinoma	c-Met	Topel et al. (2020)
lncRNA ANRIL	Glioma	miR-203	Dai et al. (2018)
lncRNA APOC1P1-3	Breast cancer	miR-188-3P/Bcl-2	Lu et al. (2021)
lncRNA H19	Colorectal cancer	miR-29-3b	Ding et al. (2018)
lncRNA FOXD2-AS1	Thyroid carcinoma	miR-7-5p/TERT	Liu et al. (2019c)
lncRNA MAPK8IP1P2	Thyroid cancer	miR-146b-3p	Liu et al. (2020b)
lncRNA NEAT1	Cervical cancer	miR-124/NF-kB	Shen et al. (2020)
lncRNA MALAT1	Ovarian cancer	RBFOX2/KIF1B	Gordon et al. (2019)
lncRNA VAL	Lung adenocarcinoma	Trim 16/vimentin	Tian et al. (2020)
LINC00958	Bladder tumor	Metadherin	Seitz et al. (2017)
lncRNA TINCR	Breast cancer	miR-7	Liu et al. (2018)

## lncRNAs promote pre-metastatic niche formation

The concept of stem cell niche was first postulated by Schofield (1978) as a distinctive microenvironment that modulates stem cell activity during hematopoiesis. Different stem cell models have well-characterized specialized cellular niches, which play an important role in balancing stem cell activity and quiescence (Scadden, 2014). In the stromal microenvironment of stem cells, it is a distinct local region that combines signals reflecting tissue and organismal state (Schofield, 1978) and modulates epithelial cell plasticity and stem cell fate commitment during tissue regeneration and homeostasis (Blanpain and Fuchs, 2014). In the context of tumor development, tumor cells subvert and shape the niche to create a compatible metastatic niche (Barcellos-Hoff et al., 2013) that supports the growth and survival of disseminated tumor cells (DTCs) and result in the development and progression of disease (Psaila and Lyden, 2009; Sleeman, 2012). Metastatic niches can be formed either on arrival of DTCs in the recipient tissue (Sleeman, 2012) or under the regulation of secreted factors and/or exosomes released by the primary tumor cells before the seeding of DTCs (also termed the pre-metastatic niche) (Kaplan et al., 2006; Hoshino et al., 2015; Peinado et al., 2017). Tumor-derived molecular components (TDMCs), often referred to as non-vesicle factors, and extracellular vesicles are adaptable intercellular communication vehicles that can modulate signaling in the TME while developing the pre-metastatic niche. TDMCs are subdivided into non-vesicle tumor-derived secreted factors (TDSFs) and tumor-derived secreted extracellular vehicles (EVs) which carry a variety of molecular components, such as RNA, DNA proteins, and lipid molecules (Liu and Cao, 2016). In recent years, ncRNAs, such as lncRNAs and sncRNAs, have been identified as an essential part of pre-metastatic niche formation and a new intercellular communication mechanism (Xie et al., 2019). These ncRNAs, as part of TDMCs, are mostly found in EVs with only a small percentage being free (Das et al., 2019). EVs have a unique repertoire of lncRNAs and sncRNAs, highlighting the importance of ncRNAs in the formation of the pre-metastatic niche (Ma et al., 2017; Pardini and Calin, 2019).

Previously, primary tumor–derived vesicles have been linked to the development of the pre-metastatic niche (Kaiser, 2016). In addition, exosomes secreted by primary tumor cells have been shown to enter the bloodstream and alter the metastatic microenvironment for invasion (Szatanek et al., 2017). Recently, lncRNAs, for example, lincROR, DREH, MALAT, CCAT2, HOTAIR, BCAR4, and H19, have been shown to contribute to metastasis *in vitro* and *in vivo* (Weidle et al., 2017). In addition to modulating the release of exosomes, lncRNAs modify the cell physiology of distant non-tumor cells and, at the pre-metastatic niche, allow the early survival of disseminate tumor cells (Liu et al., 2019a). Endothelial cells transfer and internalize lncRNA H19 in CD90^+^ HCC cells and promote angiogenesis and intracellular adhesion by enhancing the release and production of VEGF (Conigliaro et al., 2015; Zhang et al., 2019c). In Hepatocellular cancer (HCC)/liver cancer (LiC) cells, the integrin *β*1/α5/JNK/c-JUN signaling pathway participates in higher matrix stiffness, which is induced by LOXL2 (lysyl oxidase homolog 2). LOXL2 stimulates the expression of CXCL12 and MMP-9, the production of fibrin, and the recruitment of bone marrow–derived dendritic cells (BMDCs), supporting the development of the pre-metastatic niche (Wu et al., 2018). In pancreatic ductal adenocarcinoma (PDAC), exosome-derived protein macrophage migration inhibitory factor (MIF) enhances metastasis of the liver by promoting the development of the hepatic pre-metastatic niche. Additionally, the lncRNA SOX2OT has been found to regulate the expression of Sox2 *via* competitively binding with the miR-200 family, leading to the EMT process and stem cell–like characteristics, which are hallmarks of cancer metastasis (Li et al., 2018b), thereby leading to metastasis and invasion of PDAC. Researchers from the School of Public Health and Medicine University of Wisconsin, USA have proposed that cancer stem-like cells (CSCs) and anaplastic TC (ATC) release lncRNAs (HOTAIR, lincROR, MALAT1, and PVT1) transferred by the exosome and therefore help to induct EMT, modulate host immunity to escape immune response, and inculcate the TME to form a metastatic niche (Hardin et al., 2018).

## Targeting lncRNAs for the treatment of metastatic tumors

Current treatments for metastasis are similar to those for primary tumors and include immunotherapy, chemotherapy, targeted therapy, and a combination of these (Massagué and Obenauf, 2016). However, patients with metastatic cancer frequently exhibit increased therapeutic resistance in multiple carcinomas (Jolly et al., 2019), indicating that exploring new strategies for diagnosis and therapeutics is an urgent priority. The expression of certain lncRNAs is modified during transformation from primary to metastatic cancer cells, and these changes can serve as potential diagnostic biomarkers for cancer. In addition to identifying the molecular mechanisms of lncRNAs in cancer metastasis, considerable efforts should be acquired to investigate the promising strategies for clinical implications. The expression of certain lncRNAs can be detected not only in the cells themselves but also in exosomes found in the serum. One example is the lncRNA HOTAIR which has shown promise as a diagnostic marker for thyroid cancer and can differentiate benign thyroid nodules from migratory. Clinical trials are ongoing to further explore its potential use as a diagnostic tool for THC (NCT03469544) (Zhang et al., 2017b; Lai and Cheng, 2018). Early detection of lncRNA expression changes in patients can lead to personalized and precision medicine by allowing for early treatment at a time when the disease is more responsive to medication. Although direct targeting of lncRNAs as a therapeutic intervention is still in its early stages, various methods are being developed to modulate lncRNA expression, such as transcription blocking, degradation, and gene-editing technology. DNA binding elements can modulate lncRNA transcription, while techniques such as ribozymes, antisense oligonucleotides (ASOs), and siRNAs *via* activating an RNA-induced silencing complex (RISC) can downregulate lncRNAs by inducing their degradation. For instance, lncRNA MALAT1 can be efficiently silenced by zinc finger nucleases (ZFNs)–based genomic manipulation (Gutschner et al., 2011). Depletion of oncogenic lncRNAs in cancer cells has shown anti-cancer properties, such as inhibiting the proliferation and cloning of lung cancer cells. For instance, siRNA-induced KD of the lncRNA LL22NC03-N64E9.1 inhibited the proliferation of lung cancer (LC) cells (Jing et al., 2018). Likewise, intra-tumor administration of siRNA targeting lncRNA MALAT1 in a prostate cancer (PCa) xenograft model decreased metastasis and improved mice survival (Ren et al., 2013). Similarly, siRNAs targeting lncRNA OIP5-AS1 reduced migration, invasion, and proliferation of glioma U87 cells (Sun et al., 2019). siRNA mediated KD of lncRNA PCGEM1 (PCa gene expression marker 1) has been shown to increase G2- and S-phase cells, inhibit colony formation, and enhance sensitivity to baicalein in PCa LNCaP cells (Han et al., 2020). ASOs are chemically synthesized RNA-targeting molecules that range from 12 to 30 nucleotides in length. They bind to specific RNA targets through Watson–Crick base pairing (Bennett, 2019) and modulate gene expression by initiating target degradation, blocking translation, steric hindrance, altering splicing (Arun et al., 2018), and premature transcriptional termination (Lai et al., 2020; Lee and Mendell, 2020). ASOs have shown promising results as a therapeutic approach for lncRNA targeting and have been shown to slowdown tumor growth and reduce metastasis in mouse mammary cancer models (Zhou et al., 2016). In the mouse mammary tumor virus (MMTV)-PyMT cancer model, the KD of lncRNA MALAT1 by ASOs results in slower tumor growth and a reduction in metastasis (Arun et al., 2016). However, optimizing the delivery of ASOs is a crucial issue to improve their efficacy for targeting lncRNAs or protein-coding genes (Yamamoto et al., 2015; Lai et al., 2020). Recently developed miR-CLIP-seq technology is used to identify the miRNA-mRNA (Ahadi et al., 2017) and miRNA-lncRNA interactions (Cao et al., 2019). For instance, the interactions between miR-106a and lncRNA H19 and their effect on the upregulation of downstream-associated mRNAs have been studied (Imig et al., 2015).

Another method of targeting lncRNAs is through competitive binding using specific small molecules or aptamers. Aptamers are short RNA or DNA oligonucleotides that can bind to specific regions of lncRNA and block its interactions with binding partners. Small molecule inhibitors target the RNA binding pockets of lncRNAs, preventing the interactions between lncRNAs and proteins (Parasramka et al., 2016). However, the potential for off-target effects and high costs in targeting the interaction between lncRNAs and their targets require further research before these methods can be used in therapeutic applications. CRISPR/Cas9 or CRISPR/Cas13–based targeting technology is becoming increasingly important due to recent advancements in genome editing techniques, making it a promising alternative for lncRNA regulation (Cox et al., 2017; Zhen and Li, 2019). In animal models, lncRNA GMAN was successfully targeted using CRISPR/Cas9, resulting in a significant decrease in GC metastasis (Zhuo et al., 2019). Despite the fact that lncRNAs are promising therapeutic targets for metastatic diseases, their *in vivo* inhibition still remains a challenge because of their quick degradation in biological fluids by nucleases, and the difficulty of delivering them to specific target cells and the activation of innate immunity. A variety of materials are being researched to overcome these challenges, which include lipid nanoparticles (Pirollo et al., 2007), polymers (Shu et al., 2014), cell-penetrating peptides (CPPs) (Yang et al., 2015), monoclonal antibodies (Yao et al., 2012), and small-molecule inhibitors (Thomas et al., 2009). However, further research is required before these findings can be translated into clinical applications.

## Conclusion and future perspective

Metastasis is the leading cause of cancer-related deaths and a major barrier to successful cancer treatment. The complex process of metastasis is influenced by interrelated signaling pathways caused by genetic heterogeneity or epigenetic modification changes and the metastatic microenvironment. lncRNAs have gained attention in recent years due to their important role in promoting and maintaining tumor initiation and progression (Schmitt and Chang, 2016). This article focuses on the role of lncRNAs in the regulation of key processes in metastasis, which includes metabolic reprogramming (glucose metabolism and OXPHOS), EMT, metastatic microenvironment, development of pre-metastatic niches, drug resistance, and AR.

Due to their unique and diverse functions, lncRNAs have been identified as potential targets for cancer therapy. For example, lncRNAs have been found to be involved in regulating gene expression and chromatin structure, both of which are critical processes in cancer progression and metastasis. However, the majority of research on lncRNAs and metastasis to date has been concentrated on metastasis specific to organs. However, many patients suffer from metastasis in multiple organs, making it difficult to understand the molecular mechanisms mediated by lncRNAs. Because of this intricacy, it is difficult to understand scientific molecular mechanisms mediated by lncRNAs, and therefore it has been challenging to generalize the functions of lncRNAs in metastasis across different types of cancers due to the cell type–specific nature of most lncRNAs. Generalizing the functions of lncRNAs in metastasis across different types of cancers has also been elusive, and perhaps this should be expected based on the cell type–specific function of most lncRNAs. The contribution of lncRNAs to the steps in the invasion–metastasis cascade that includes intravasation, extravasation, distant colonization, and formation of micrometastases is not well established. Therefore, more patient-matched molecular profile data from granular stages of metastasis are necessary to overcome these limitations (for example, CTCs, micrometastases, primary tumor, and well-defined metastases).

lncRNAs appear to be an underappreciated cache of novel therapeutic targets. They play a variety of roles in the progression of cancer and ensure new opportunities for undermining metastases in the clinical setting. Thus, future research may focus on identifying lncRNA mediators of metastasis as potential targeted therapies common in various types of tumors. The development of lncRNA-based therapeutics requires the identification and validation of specific lncRNA targets involved in metastasis. To achieve this, researchers have to develop reliable technologies that are capable of targeting lncRNAs *in vivo* (Bassett et al., 2014; Liu and Lim, 2018), such as antisense oligonucleotides (Wurster and Ludolph, 2018) that can specifically bind to lncRNAs and modulate their expression. The availability of these technologies will significantly facilitate the development of lncRNA-based therapeutics for the treatment of metastatic cancer. However, there are still many challenges in lncRNA research, such as the lack of conservation of lncRNAs across species, which can hinder the study of their function in animal models, complexity and heterogeneity in function, and tissue specificity. Furthermore, there is a requirement for more sensitive and specific technologies to effectively target lncRNAs *in vivo*. Despite these challenges, the potential of lncRNAs as therapeutic targets in cancer is evident, and future research in this area has the potential to impact the successful treatment of metastatic cancer.

## Figures and Tables

**Table:** 

**ATP**	adenosine triphosphate
**ASOs**	antisense oligonucleotides
**AMPK**	adenosine monophosphate–activated protein kinase
**BNIP3**	BCL2/adenovirus E1B 19 kDa interacting protein 3
**ceRNA**	competing endogenous RNA
**CSC**	cancer stem-like cells
**DTCs**	disseminated tumor cells
**EVs**	extracellular vesicles
**EMT**	epithelial–mesenchymal transition
**EGFR**	epidermal growth factor receptor
**GDH**	glutamate dehydrogenase
**GBC**	gallbladder cancer
**GLUT**	glucose transporter
**HKs**	hexokinases
**HIF**	hypoxia-inducible factor
**hnRNPL**	heterogeneous nuclear ribonucleoprotein L
**LncRNAs**	long non-coding RNAs
**LDHA**	lactate dehydrogenase A
**LKB1**	tumor suppressor liver kinase B1
**mTOR**	mammalian target of rapamycin
**MMP**	matrix metalloproteinase
**MIF**	macrophage migration inhibitory factor
**ncRNAs**	non-coding RNAs
**NANOG**	non-processed pseudogene
**NF-κB**	nuclear factor-kappa B
**piRNAs**	piwi-interacting RNAs
**PKM2**	pyruvate kinase M2
**PCGs**	protein-coding genes
**PFKFB2**	6-phosphofructo-2-kinase/fructose-2,6-biphosphatase 2
**PI3K**	phosphoinositide 3-kinase
**PAX8**	paired-box gene 8
**PDAC**	pancreatic ductal adenocarcinoma
**rRNA**	ribosomal RNA
**ROS**	reactive oxygen species
**siRNAs**	small interfering RNAs
**SOX**	SRY box transcription factor
**STAT3**	signal transducer and activator of transcription 3
**tRNA**	transfer RNA
**TME**	tumor microenvironment
**TGIF2**	TGF (transforming growth factor) β–induced factor homeobox 2
**TNF-α**	tumor necrosis factor-α
**TDMCs**	tumor-derived molecular components
**TERT**	telomerase reverse transcriptase
**TGF-β**	tumor growth factor beta
**VEGF**	vascular endothelial growth factor
**WBC**	white blood cells
